# Plutonium and thorium isotopes in the bottom sediments of some Mazurian Lakes (Poland)

**DOI:** 10.1007/s10967-018-6300-8

**Published:** 2018-11-02

**Authors:** J. W. Mietelski, Jolanta Wojtycza, Marek Zalewski, Jacek Kapała, Ewa Tomankiewicz, Paweł Gaca

**Affiliations:** 1 Institute of Nuclear Physics Polish Academy of Sciences, Radzikowskiego 152, 31-342 Kraków, Poland; 2 Marshal Office of the Małopolska Region, Racławicka 56, 30-017 Kraków, Poland; 3 Department of Biophysics, Medical Academy of Białystok, Mickiewicza 2a, 15-089 Białystok, Poland; 4 GAU-Radioanalytical Laboratories, Ocean and Earth Science, University of Southampton, National Oceanography Centre, European Way, Southampton, UK

**Keywords:** Pu in environment, Radionuclides in lake sediments, Mazurian Lakes, Chernobyl, Global fallout

## Abstract

Presented are results on the Pu and Th isotopes activity concentration found in the upper part of bottom sediments collected from a deep part of 29 lakes in N-E Poland by diving in 2000. Analyses of Pu isotopic ratios allowed for the discussion of Pu origin. Maximum percentage of ^239+240^Pu activity from Chernobyl fallout was 24%. Surface deposition of ^239+240^Pu was calculated. No relationship was found between the plutonium and main chemical matrix components of sample nor with the trophic status of the lake. Pu activities were weakly correlated with measured previously ^137^Cs activities.

## Introduction

Study on the plutonium concentration in forest litter [1–3] conducted on the area of North-Eastern Poland, especially in Augustów Primeval Forest, showed the significant deposition of Chernobyl plutonium on some areas there. The activities were up to 30 Bq/m^2^ for alpha emitting isotopes: ^238+239+240^Pu [1] and up to 1 kBq/m^2^ (in 1986) for ^241^Pu [2]. Deposition of Pu isotopes from Chernobyl most likely took place in a form of small “fuel like hot particle” and therefore by nature was rather non homogeneous. Earlier deposition of ^239+240^Pu alpha emitters from global fallout [4] was in average equal to about 60 Bq/m^2^, and remains of that for relatively short living (*T*_1/2_ = 14.4 a) beta emitter, the ^241^Pu was equal to about 100 Bq/m^2^ in year 2001. Global deposition of ^238^Pu from SNAP 9A satellite accidental re-entry in 1964 was small [4], for our latitude it was equal to about 2.5 Bq/m^2^.

One of the aims of present work was to get some data on Pu activity in examined upper part of bottom sediments for which radiocaesium data were obtained [5]. It seemed for authors interesting to get general recognition on level of Pu present in Mazurian lakes deep sediments for any further analyses in sediments of shallow waters. Another aim was to compare the data obtained from analyses conducted in the forest environment with the results from lake sediments. It seemed interesting for us also to complete the results of Pu and Th isotopes to those for radiocaesium [5] obtained before for the same bottom sediment samples.

## Experimental

### Samples

Samples were collected during free diving by one of us (Marek Zalewski) in May 2000. A year later he sadly passed away and this work was no published till today. Now we want to commemorate his Person finishing the idea which he started.

**Fig. 1:**
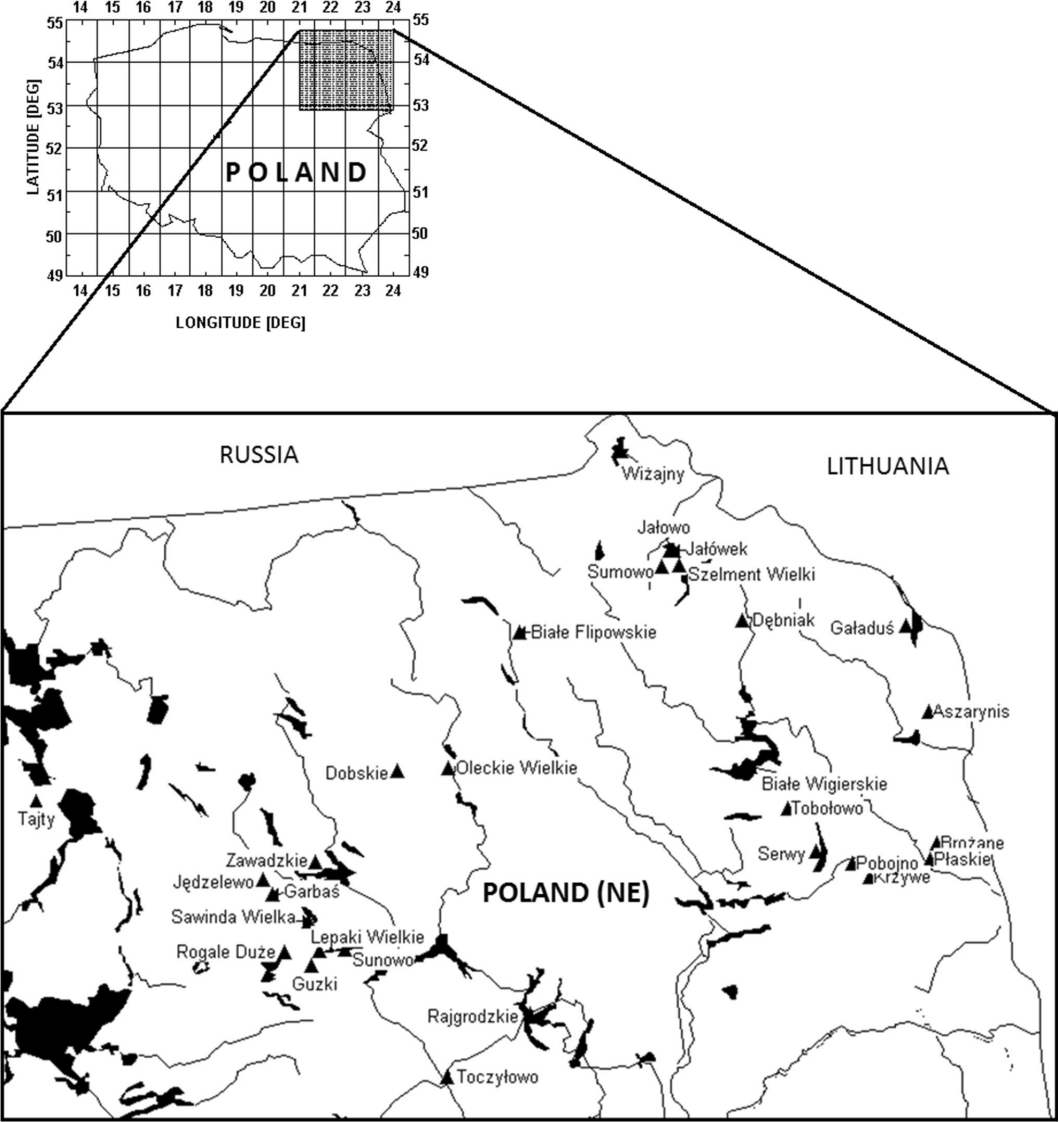
Location of the lakes on the map of Poland

Samples of bottom sediments were taken from 29 lakes in North-Eastern Poland. The 17 lakes were eutrophic [6] (Brożane, Dębniak, Guzki, Jałówek, Jędzelewo, Krzywe, Lepaki, Oleckie, Pobojno, Rajgrodzkie, Rogale, Sawinda Wielka, Tajty, Tobołowo, Toczyłowo, Wiżajny, Zawadzkie), 9 were mesotrophic lakes [6] (Dobskie, Gaładuś, Garbaś, Jałowo, Płaskie, Serwy, Sumowo, Sunnowo, Szelment Duży) and the 3 were oligotrophic lakes [6] (Aszarynis, Białe Filipowskie, Białe Wigierskie). In one case (Sumowo) two samples were taken in different parts of lake. All lakes are lying in the Eastern part of Mazurian lakes area. A map of North-Eastern Poland showing the lakes locations is presented in Fig. 1. Some data characterizing the lakes are presented in Table 1. The lakes have different hydrological status [6]. Six of them are endoreic (landlocked) lakes, 12 are transit lakes (i.e. there is at least one river coming in and one going out the lake) and 11 are outflow lakes (i.e. there is no river coming in). More details on the lakes themselves might be found in the monographic publication on Polish lakes [6]. To make it more easy the code numbers given to the lakes in that book are also presented in Table 1.

**Table 1:** Some data on examined lakes [5, 6]

Name of lake	Code in [5]	Main surrounding of lake	Trophic status	pH	Ca conc. (mgCa/dm^3^)	Hydro-logical status	Drain Basin area (ha)	Lake area (ha)	Water volume (10^3^ m^3^)	Max. depth (m)	Mean depth (m)
Aszarynis	682	F	O	8	8.6	Endoreic	3.8	12.6	454	7.8	3.6
Białe Filipowskie	583	A	O	8.3	33.6	Endoreic	4.8	132.4	22,662	52	17.1
Białe Wigierskie	622	F	O	8.3	30.7	Outflow	2	100.2	13,194	34	13.2
Brożane	651	F	E	8.3	47.8	Transit	27.4	44.6	2998	24.3	6.7
Dębniak	–	F	E	–	–	Outflow	0.5	6.6	0.26	7.7	3.9
Dobskie	75	A	M	8.4	39.3	Transit	108	162.5	18,026	43.3	11.1
Gaładuś	611	A	M	8.7	42.8	Outflow	85.5	728.6	92,475	54.8	12.7
Garbaś	105	F	M	8.8	40.7	Outflow	6.2	42.5	4343	38	10.2
Guzki	119	A	E	8.7	48.6	Transit	1.2	23.3	865	6	3.7
Jałówek	–	A	E	–	–	Outflow	–	–	–	–	–
Jałowo	602	A	M	8.3	46.4	Endoreic	–	20.1	1367	16.1	6.8
Jędzelewo	102	F	E	8.7	63.5	Transit	213.9	150.3	4967	13.1	3.2
Krzywe	657	F	E	8.2	52.8	Transit	–	21.6	499	6	2.3
Lepaki Wielkie	120	A	E	8.3	69.3	Transit	4.8	19.7	808	8	4.1
Oleckie Wielkie	45	A	E	8	56.4	Transit	124.8	227.3	37,912	45.2	16.7
Płaskie	650	F	M	8.2	35.7	Endoreic	2.3	56.8	2854	12	5
Pobojno	655	F	E	8.4	62.1	Endoreic	4	23.6	716	6.2	3
Rajgrodzkie	52	F	E	8	57.8	Artifitial transit	696.4	1503.2	142,623	52	9.4
Rogale Duże	246	A	E	8.5	48.8	Outflow	3.8	31.7	2275	22.1	7.2
Sawinda Wielka	115	F	E	8.5	70	Outflow	34.1	224.8	10,554	9.5	4.7
Serwy	652	A	M	8.6	41.7	Artifitial outflow	54.7	460.3	67,182	41.5	14.6
Sumowo	601	A	M	8.1	52.1	Transit	190.5	30	662	8	2.2
Sunnowo	118	A	M	8.3	50.7	Transit	73.3	176.3	16,456	20.6	9.3
Szelment Wielki	606	A	M	8.3	46.4	Outflow	54.1	356.1	53,492	45	15
Tajty	131	F	E	8.6	48.6	Transit	–	265.1	19,826	34	7.5
Tobołowo	24	F	E	8.3	37.1	Endoreic	17.4	51.4	2107	9.4	4.1
Toczyłowo	124	F	E	8.7	78.5	Artifitial. Outflow.	60	101.8	4864	9.9	4.8
Wiżajny	579	A	E	7.9	31.4	Transit	13.4	293.1	7746	5.3	2.6
Zawadzkie	91	A	E	8.3	50	Outflow	7.6	96.2	2605	9.7	2.7

*A* agricultural, *F* forests, *E* eutrophic, *M* mesotrophic, *O* oligotrophic

Sediments were taken in central part of each lake, off-site of littoral zone, where on bottom no vegetation was observed [5]. Diving were conducted till the depth enabling visibility sufficient for sample collection. For oligotrophic lakes it was nearly 20 m. In other cases it was less, not more than 10 m. Sediments were collected and taken to the surface as monolithic cores in plastic tubes of 10 cm diameter and 30 cm height [5]. In given site sampling repetitions nearby (from few to dozens of meters) were done. Cylinders were pressed into the bottom sediment, blocked from both ends and then taken out to the water surface. In majority of cases collected material had a form of a sludge. Samples were air-dried in laboratory for several weeks until they become a dry. Shrank sediment samples were sliced into 2 cm height layers, homogenised and taken for gamma spectrometric measurements [5]. The contraction factor (ratio of original total sample height to height of dried material) varied from 2 to 2.8. For 19 lakes only top 2 cm were taken. After performing analyses for all 29 lakes the 10 lakes for which additional profile taken close to first one was available (Białe Wigierskie, Gaładuś, Garbaś, Guzki, Płaskie, Rogale Duże, Sawinda Wielka, Sumowo, Toczyłowo, Zawadzkie) we did repeated analyses for plutonium using more than one layer. Those samples were also sliced in 2 cm thick layers. Typically, three slices were obtained, however in two cases collected material allowed to use only two samples and in one case four layers were obtained. This repeated analyses were performed for determination of alpha emitting plutonium isotopes only. All presented data refers to dried material. For quality assurance a reference material IAEA 300 Baltic Sea sediment [7] was analysed as well.

### Procedures and measurements

Applied procedure for Pu and Th determination was an our implementation [1, 3] of the radiochemical method [8] used by J.J. LaRosa and his co-workers for the analyses of Chernobyl Project samples in the IAEA Laboratories at Seibersdorf. The procedure started with incineration in 600 °C lasting for at least for 8 h. Ten gram ash was taken from each sample for further radiochemical procedure. The plutonium tracers (^236^Pu of 9.3 mBq activity at 1.03.2001 in first set and 3.4 mBq of ^242^Pu in case of repetited samples) was added to each sample and subsequent wet mineralization in hot concentrated acids (HF, HNO_3_, HCl + H_3_BO_3_) was conducted. The oxidation adjustment procedure [1, 8] was performed using hydrazine and NaNO_2_. Thorium and plutonium was separated by anion-exchange chromatography using DOWEX 1 × 8, 200 mesh, Cl^−^ form (Sigma Ltd.). Thorium was stripped off the column with 10 M HCl. Plutonium was later striped off the column with 0.1 M HCl–0.1 M HF solution. Thin alpha spectrometric sources were prepared form both Th and Pu stripes using NdF_3_ co-precipitation method [8, 9] using 50 nm pore diameter filters.

The alpha spectrometric measurements were performed using Silena AlphaQuattro alpha-spectrometer with silicon detectors (Canberra PIPS, 450 mm^2^). Alpha-sources were measured in about 2 mm source to detector distance. Obtained spectra were analysed using home made ALF software [1]. Mean value of FWHM was equal to 68 keV with standard deviation of 16 keV.

In the aim of the determination of ^241^Pu activity Pu alpha-sources were then dissolved in small amounts of hot concentrated HNO_3_ with traces of H_3_BO_3_ added, evaporated to dryness, re-dissolved in 1 M HNO_3_, mixed with liquid scintillation cocktail Wallac HiSafe3 and measured for the ^241^Pu using Wallac 1414-003 Guardian liquid scintillation spectrometer. More details on procedure was presented previously [2]. The spectrometer can distinguish [10] scintillations caused by alpha particles from those caused by beta particles and therefore a simultaneous separate acquisition of alpha and beta spectra is possible. The total alpha emitting Pu isotopes activity present in source, which was already known here as the result of the alpha spectrometric measurement, was used to control the recovery. The tritium protocol [2, 11] together with built-up library data was applied for all quenching and efficiency problems. Details of applied method of ^241^Pu activity calculation were described already elsewhere [2]. Measurement time was set for 10^5^ s.

### Calculation of Chernobyl component

The analysis of plutonium activity concentration ratios (^238^Pu to ^239,240^Pu) allowed us to distinguish the Chernobyl and global fallout components of plutonium activities. Assumed ^238^Pu to ^239,240^Pu activity ratio in global fallout was equal to 0.03, and that for Chernobyl fallout was taken equal to 0.50 (decay corrected for year 2000), from 0.55 in fresh fallout, the percentage *F*_o_ of Chernobyl plutonium can be calculated [1] as follow:

1 $$F_{\text{o}} = \left( {\frac{{A_{238} /A_{239} - 0.03}}{0.50 - 0.03}} \right) \cdot 100\%$$

where *A*_238_—activity concentration of ^238^Pu, *A*_239_—activity concentration of ^239,240^Pu.

Global fallout component is equal to 100%—*F*_o_.

The analysis of another plutonium activity concentration ratios, ^241^Pu to ^239,240^Pu also allows to distinguish the Chernobyl and global fallout components of plutonium activities. The ^241^Pu to ^239,240^Pu activity ratio in global fallout [4] was equal to about 12 in 1963, and that for Chernobyl fallout was equal 86 [2] in 1986. Corrected for the decay, at the year 2001 those ratio should be equal to 1.7 and 41.7, for global and Chernobyl fallouts, respectively. Thus, the percentage *F*_1_ of Chernobyl plutonium based on ^241^Pu activity can be calculated as follow:

2 $$F_{1} = \left( {\frac{{A_{241} /A_{239} - 1.7}}{41.7 - 1.7}} \right) \cdot 100\%$$

where *A*_241_—activity concentration of ^241^Pu, *A*_239_—activity concentration of ^239,240^Pu.

Global fallout component is equal to 100%—*F*_1_.

### Calculation of surface deposition

The surface activities *A*_surf_ in sediments were calculated from the activity concentrations *A*_conc_ for radiocesium and then plutonium accordingly to the following formula:

3 $$A_{\text{surf}} = A_{\text{conc}} V\;\rho \;S^{ - 1}$$

where *ρ*—density for dry material; *V*—volume of dry mass taken for analyses; *S*—surface area of a sampling cylinder.

## Results and discussion

Results on ^239+240^Pu and ^238^Pu activity concentration (calculated for summer 2001) for initial set of uppermost ttop 2 cm of sediments are presented in Table 2. The last row of Table 2 contains results for the Reference Material (RM) IAEA 300. Obtained value for ^239+240^Pu activity in RM of 3474 ± 262 mBq/kg is close to certified value [7] 3550 mBq/kg and it lies within the confidential limits from 3440 to 3650 mBq/kg. For samples the ^239+240^Pu range from 81 ± 13 mBq/kg for sediment of Dębniak Lake to 3820 ± 265 mBq/kg for that of Zawadzkie Lake, whereas for ^238^Pu they range from < 13 mBq/kg for Dębniak (again minimum value) or 9 ± 5 mBq/kg for Rogale Duże Lake up to 549 ± 122 for Zawadzkie Lake (again maximum value). Arithmetic mean activity for ^239+240^Pu was 1285 mBq/kg with the standard deviation (SD) equal to 1012 mBq/kg. Relatively higher SD value compare to mean value was observed for ^238^Pu, mean was 72 mBq/kg and SD = 97 mBq/kg. Arithmetic mean for recovery was 83.8% with SD equal to 14.5%. The last column of Table 2 contain results for surface activity of ^239+240^Pu in examined samples. In one third of samples observed plutonium deposition in examined layer is low, below 10 Bq/m^2^. For no sample the deposition exceeds significantly 60 Bq/m^2^, however in some cases it is very close to this value. The men value is 19 Bq/m^2^ with standard deviation of 15 Bq/m^2^. This suggested, that majority (60–70%) of plutonium was situated deeper in the sediment profiles. This was in a good consistency with results for Swedish lakes [12], where the maximum of Pu deposition was at about 3 cm, and whole deposit was in fist 6 cm of sediment. The variety of plutonium deposition observed in examined surface layer samples of bottom sediments reflects most likely the differences in the sediment accumulation rate, which depends on several parameters [13]. Results are lower than those for Obleije and Żuwintas lakes in Southern Lithuania [17] where maximum activities of 7500 ± 400 mBq/kg and 1800 ± 500 mBq/kg were found for ^239+240^Pu and ^238^Pu, respectively.

**Table 2:** Results on ^239+240^Pu and ^238^Pu activity concentration (calculated for summer 2001) in top 2 cm bottom sediments of investigated lakes

Name	Dry mass (g)	Recovery (%)	^239+240^Pu (mBq/kg)	^238^Pu (mBq/kg)	^239+240^Pu (Bq/m^2^)
		Y ± dY	A ± dA	A ± dA	A ± dA
Aszarynis	12.4	73.8 ± 3.3	178 ± 24	< 19	3.0 ± 0.4
Białe Filipowskie	11.2	74.8 ± 6.3	2738 ± 168	115 ± 58	33.1 ± 2.0
Białe Wigierskie	16.0	27.7 ± 2.5	2470 ± 272	61 ± 18	30.3 ± 3.3
Brożane	11.5	93.6 ± 4.4	414 ± 37	< 35	8.7 ± 0.8
Dębniak	10.9	100.5 ± 6.0	81 ± 13	< 15	1.1 ± 0.2
Dobskie	11.7	91.7 ± 4.2	1004 ± 71	64 ± 21	21.9 ± 1.5
Gaładuś	12.7	98.9 ± 4.4	2746 ± 163	90 ± 18	60.4 ± 3.6
Garbaś	11.1	78.3 ± 5.1	2849 ± 167	82 ± 36	50.8 ± 3.0
Guzki	12.7	69.5 ± 4.8	1697 ± 109	< 90	25.2 ± 1.6
Jałówek	18.7	94.6 ± 5.0	776 ± 53	33 ± 19	10.9 ± 0.7
Jałowo	11.2	71.7 ± 3.8	485 ± 45	< 50	5.9 ± 0.5
Jędzelewo	12.8	61.8 ± 3.5	572 ± 48	53 ± 28	6.8 ± 0.6
Krzywe	13.1	94.3 ± 4.8	1465 ± 97	44 ± 18	18.1 ± 1.2
Lepaki Wielkie	10.5	91.2 ± 4.1	238 ± 30	16 ± 14	4.0 ± 0.5
Oleckie Wielkie	12.8	79.3 ± 6.9	761 ± 72	< 90	12.1 ± 1.1
Płaskie	13.5	81.2 ± 4.0	564 ± 45	< 50	7.9 ± 0.6
Pobojno	15.1	86.6 ± 3.8	730 ± 49	70 ± 17	19.4 ± 1.3
Rajgrodzkie	11.3	100.8 ± 4.7	186 ± 27	< 20	4.2 ± 0.6
Rogale Duże	10.3	93.1 ± 3.7	224 ± 24	< 15	3.0 ± 0.3
Sawinda Wielka	13.8	90.8 ± 4.4	1177 ± 74	34 ± 17	10.0 ± 0.6
Serwy	13.8	89.9 ± 4.0	1575 ± 97	60 ± 16	18.7 ± 1.1
Sumowo 1	13.1	97.0 ± 6.2	1919 ± 117	68 ± 30	30.4 ± 1.8
Sumowo 2	13.8	83.1 ± 6.6	1747 ± 110	161 ± 44	33.2 ± 2.1
Sunnowo	12.7	76.6 ± 4.2	1650 ± 106	86 ± 27	20.9 ± 1.3
Szelment Wielki	11.3	94.6 ± 4.6	576 ± 41	44 ± 19	9.1 ± 0.6
Tajty	12.6	85.3 ± 4.0	2976 ± 205	106 ± 14	28.3 ± 1.9
Tobołowo	11.9	90.6 ± 5.2	535 ± 46	24 ± 8	6.5 ± 0.6
Toczyłowo	11.8	66.8 ± 5.5	1806 ± 117	< 100	18.2 ± 1.2
Wiżajny	20.2	88.2 ± 4.6	585 ± 44	24 ± 12	10.4 ± 0.8
Zawadzkie	13.4	83.7 ± 4.5	3819 ± 265	549 ± 122	52.2 ± 3.6
IAEA 300*	5	87.0 ± 5.4	3474 ± 262	164 ± 75	

The last column presents results for surface deposition of ^239+240^Pu. The last row contains results for the Reference Material IAEA 300 obtained with ^236^Pu tracer. Uncertainties are 1*σ* counting statistic only

*Reference Material, certified value [7]: 3550 (from 3440 to 3650) mBq/kg

Regarding ^241^Pu measurements only in one case, for Zawadzkie Lake, the ^241^Pu was measurable by LSC without doubts, and the activity was equal to 50.5 ± 3.2 Bq/kg. Another result above the detection limit is for Garbaś Lake, where it was 5.3 ± 4.7 Bq/kg with the minimum detectable activity of 2.2 Bq/kg. In all other cases the activities were below the detection limits, which varied from 2 to 6 Bq/kg.

Since our initial study was limited to the top layer ony because of the amount of ^236^Pu tracer present in laboratory after about three years we returned to the analyses using new tracer, the ^242^Pu by NIST. We suspected that subsequent amount of plutonium can be present in deeper layers. The check this a repetition run of Pu measurements was performed for 10 lake sediments, for which we had spare profiles. Results are presented in Table 3. Again, the last row contains results for RM IAEA-300 obtained during this run. Our result is 3400 ± The minimum value for ^239+240^Pu was found in depth 6–8 cm in Rogale Duże lake (9 ± 7 mBq/kg) whereas maximum was found for top 2 cm of the same lake (3670 ± 350 mBq/kg) In general those results did not confirmed results obtained for the same lakes for samples collected in the same time, when we analysed only uppermost 2 cm. It means, that very large variety of Pu content can be observed in deep sediments at distance of few meters only. Perhaps the sediments were so much not settled that the diving itself makes disturbance to it. The results confirmed that subsequent plutonium activity is present in deeper layers. In all cases but the lake Garbaś, where majority of Pu was found in top 2 cm. However in this lake the activity concentration in top leayer of second set is more than five times lower that it was found in first run.

**Table 3:** Results on ^239+240^Pu and ^238^Pu activity concentrations for second set of samples (calculated for summer 2001)

	Layer depth (contracted) (cm)	Recovery (%)	^239+240^Pu (mBq/kg)	^238^Pu (mBq/kg)	^239+240^Pu (Bq/m^2^)
Białe Wigierskie	0–2	78 ± 4	662 ± 75	29 ± 12	7.0 ± 0.8
Białe Wigierskie	2–4	89 ± 4	1118 ± 103	30 ± 10	12.0 ± 1.1
Gaładuś	0–2	74 ± 4	150 ± 25	23 ± 11	3.6 ± 0.6
Gaładuś	2–4	> 93	157 ± 18	< 12	3.6 ± 0.4
Gaładuś	4–6	> 99	172 ± 19	17 ± 6	3.8 ± 0.4
Garbaś	0–2	15 ± 1	480 ± 98	< 26	4.0 ± 0.8
Garbaś	2–4	94 ± 4	98 ± 13	6 ± 5	< 0.9
Garbaś	4–6	56 ± 3	13 ± 6	< 13	0.1 ± 0.1
Guzki	0–2	97 ± 5	1129 ± 99	181 ± 23	12.9 ± 1.1
Guzki	2–4	111 ± 6	1079 ± 94	39 ± 12	13.2 ± 1.1
Guzki	4–6	97 ± 6	722 ± 76	< 106	9.0 ± 0.9
Płaskie	0–2	> 98	812 ± 82	27 ± 12	7.1 ± 0.7
Płaskie	2–4	111 ± 9	226 ± 46	10 ± 10	1.7 ± 0.4
Płaskie	4–6	79 ± 4	29 ± 11	8 ± 8	0.2 ± 0.1
Rogale Duże	0–2	64 ± 5	3668 ± 348	166 ± 43	43.4 ± 4.1
Rogale Duże	2–4	72 ± 5	177 ± 37	16 ± 10	2.5 ± 0.5
Rogale Duże	4–6	80 ± 5	1381 ± 128	12 ± 7	19.5 ± 1.8
Rogale Duże	6–8	89 ± 5	9 ± 7	< 2	0.1 ± 0.1
Sawinda Wielka	0–2	86 ± 6	1276 ± 130	48 ± 15	9.4 ± 1.0
Sawinda Wielka	2–4	> 96	613 ± 61	54 ± 13	5.0 ± 0.5
Sumowo	0–2	116 ± 7	893 ± 95	33 ± 10	7.1 ± 0.8
Sumowo	2–4	> 98	1369 ± 110	35 ± 10	11.7 ± 0.9
Sumowo	4–6	93 ± 4	1691 ± 120	48 ± 13	14.0 ± 1.0
Toczyłowo	0–2	99 ± 5	95 ± 18	9 ± 6	1.8 ± 0.3
Toczyłowo	2–4	88 ± 5	142 ± 31	12 ± 19	2.7 ± 0.6
Toczyłowo	4–6	92 ± 5	141 ± 22	< 14	2.7 ± 0.4
Zawadzkie	0–2	90 ± 4	21 ± 7	11 ± 9	0.4 ± 0.1
Zawadzkie	2–4	93 ± 7	32 ± 9	12 ± 7	0.6 ± 0.2
Zawadzkie	4–6	60 ± 3	102 ± 18	< 3	1.9 ± 0.3
IAEA 300*		99 ± 7	3400 ± 320	141 ± 53	

The last row contains results for the Reference Material IAEA 300 obtained with ^242^Pu tracer. Uncertainties are 1*σ* counting statistic only

*Baltic Sea sediment, certified value [7] for ^239+240^Pu is 3550 mBq/kg (range 3440–3650 mBq/kg)

Table 4 shows the activity ratio of ^238^Pu to ^239+240^Pu and ^241^Pu to ^239+240^Pu as well as the estimated percentages *F*_o_ (see Eq. 1) and *F*_1_ (see Eq. 2) of Chernobyl-origin Pu in samples obtained from first analyses. Maximum ^238^Pu to ^239+240^Pu activity ratio does not exceed 15% (Zawadzkie Lake), what yields in *F*_o_= (24 ± 2) %. Such percentage of Pu Chernobyl component in this sample was confirmed by the result from ^241^Pu analyses, where *F*_1_= (29 ± 3) %. For 3 other samples Chernobyl component was about 15%, for two another it was between 5 and 10%. For those of remains samples, for which it was possible to calculate *F*_o_ value, it was below 5%. General not high content of Chernobyl fallout was rather surprising for us and perhaps unexpected, especially if one would compare present results to those for the forest litter samples, in which up to 100% of Chernobyl fraction was observed in surface layer of forest litter collected in North-Eastern Poland [1]. Such sort of discrepancy between our results in two environments suggest, that hot particles which contained Pu from Chernobyl were somehow eliminated from the examined part of sediment, or rather even never were there. One of possible way of elimination is a relatively high velocity of migration down the profile, similar to that found for forest soil from Poland [3, 14]. In the case of forest soil it was explained by a greater diameter of particles which makes them more mobile with water flow downward. Another, and even more possible in our opinion explanation of lower than expected levels of Chernobyl plutonium is the difference in the aerodynamic properties of tree canopies and lake which might influence the intensity of hot particle fallout on different surfaces.

**Table 4:** Plutonium activity ratios: ^238^Pu to ^239+240^Pu and ^241^Pu to ^239+240^Pu as well as the resulting Chernobyl-origin fractions *F*_o_ and *F*_1_ (Eqs. 2 and 3, respectively)

	^238^Pu/^239+240^Pu (%)	^241^Pu:^239+240^Pu	*F*_o_ (%)	*F*_1_ (%)	*F*_o_ */F*_1_
Aszarynis	n.d.	n.d.	n.d.	n.d.	n.d.
Białe Filipowskie	4.2 ± 2.1	n.d.	2.6 ± 2.3	n.d.	n.d.
Białe Wigierskie	2.4 ± 0.8	n.d.	n.d.	n.d.	n.d.
Brożane	n.d.	n.d	n.d.	n.d.	n.d.
Dębniak	n.d.	n.d.	n.d.	n.d.	n.d.
Dobskie	6.3 ± 2.2	n.d.	7.2 ± 1.5	n.d.	n.d.
Gaładuś	3.3 ± 0.7	n.d.	0.6 ± 0.3	n.d.	n.d.
Garbaś	2.9 ± 1.3	1.9 ± 1.7	0.0 ± 1.2	0 ± 4	n.d.
Guzki	n.d.	n.d.	n.d.	n.d.	n.d.
Jałówek	4.3 ± 2.5	n.d.	2.7 ± 3.0	n.d.	n.d.
Jałowo	n.d.	n.d.	n.d.	n.d.	n.d.
Jędzelewo	9.2 ± 4.9	n.d.	13.3 ± 5.6	n.d.	n.d.
Krzywe	3.0 ± 1.2	n.d.	0.0 ± 1.1	n.d.	n.d.
Lepaki Wielkie	6.8 ± 6.0	n.d.	8 ± 11	n.d.	n.d.
Oleckie Wielkie		n.d.	n.d.	n.d.	n.d.
Płaskie	n.d.	n.d.	n.d.	n.d.	n.d.
Pobojno	9.6 ± 2.4	n.d.	14.0 ± 1.2	n.d.	n.d.
Rajgrodzkie	n.d.	n.d.	n.d.	n.d.	n.d.
Rogale Duże	n.d.	n.d.	n.d.	n.d.	n.d.
Sawinda Wielka	2.9 ± 1.5	n.d.	0 ± 1.5	n.d.	n.d.
Serwy	3.8 ± 1.0	n.d.	1.7 ± 0.6	n.d.	n.d.
Sumowo 1	3.5 ± 1.6	n.d.	1.2 ± 1.5	n.d.	n.d.
Sumowo2	9.2 ± 2.6	n.d.	13.2 ± 1.5	n.d.	n.d.
Sunowo	5.2 ± 1.7	n.d.	4.7 ± 1.1	n.d.	n.d.
Szelment Wielki	7.6 ± 3.3	n.d.	9.9 ± 3.1	n.d.	n.d.
Tajty	3.5 ± 0.5	n.d.	1.2 ± 0.1	n.d.	n.d.
Tobołowo	4.5 ± 1.5	n.d.	3 ± 1	n.d.	n.d.
Toczyłowo	n.d.	n.d.	n.d.	n.d.	n.d.
Wiżajny	4.0 ± 2.1	n.d.	2.3 ± 2.2	n.d.	n.d.
Zawadzkie	14.4 ± 3.4	13.2 ± 2.1	24 ± 2	29 ± 3	1.2 ± 0.2

*n.d.* not determined due to result(s) below MDC (minimum detectable concentration)

Comparison of total and Chernobyl-origin inventory found for first 2 cm layer during first analyses and for deeper profile is presented in Table 5. This comparison shows clearly lack of reproducibility of results obtained. There is some similarity of results only in case of three lakes (Sumowo, Sawinda Wielka, Płaskie). In five cases the inventory of Pu found in top 2 cm is larger than then found in whole deeper profile. This shows problem related to inhomogeneity of sediments which has form of not well settled sludge. In particular, relatively high content of Chernobyl origin Pu in lake Zawadzkie was not confirmed. It seems that study should be repeated with less disturbing core sampling procedure (not man-diving but core sampler) and with at least five repetitions of sampling from one location.

**Table 5:** Inventory of ^239+240^Pu of total and Chernobyl-origin obtained for 10 lakes for which two runs were performed in course of first analyses in only top 2 cm (I) and in repeated run (II), where profile was typically analysed down to 6 cm (see text for details)

Name	Total (Bq/m^2^)	Chernobyl (Bq/m^2^)	Total (Bq/m^2^)	Chernobyl (Bq/m^2^)
	I	I	II	II
Białe Wigierskie	33.1 ± 2.0	0.9 ± 1.1	19.0 ± 1.4	0.2 ± 0.6
Gaładuś	60.4 ± 3.6	0.4 ± 0.3	11.0 ± 0.8	1.5 ± 0.9
Garbaś	50.8 ± 3.0	0	4.9 ± 0.8	0.2 ± 0.6
Guzki	25.2 ± 1.6	0	35.0 ± 1.9	3.7 ± 1.1
Płaskie	7.9 ± 0.6	0	9.1 ± 0.8	0.2 ± 0.4
Rogale Duże	3.0 ± 0.3	0	65.5 ± 4.5	1.7 ± 2.0
Sawinda Wlk.	10.0 ± 0.6	0	14.4 ± 1.1	0.8 ± 0.5
Sumowo	30.4 ± 1.8	0.4 ± 0.6	32.8 ± 1.6	0.1 ± 0.7
	33.2 ± 2.1	4.4 ± 0.7		
Toczyłowo	18.2 ± 1.2	0.5 ± 0.3	7.1 ± 0.8	0.6 ± 0.9
Zawadzkie	52.2 ± 3.6	12.5 ± 1.5	3.0 ± 0.4	0.9 ± 0.6

**Fig. 2:**
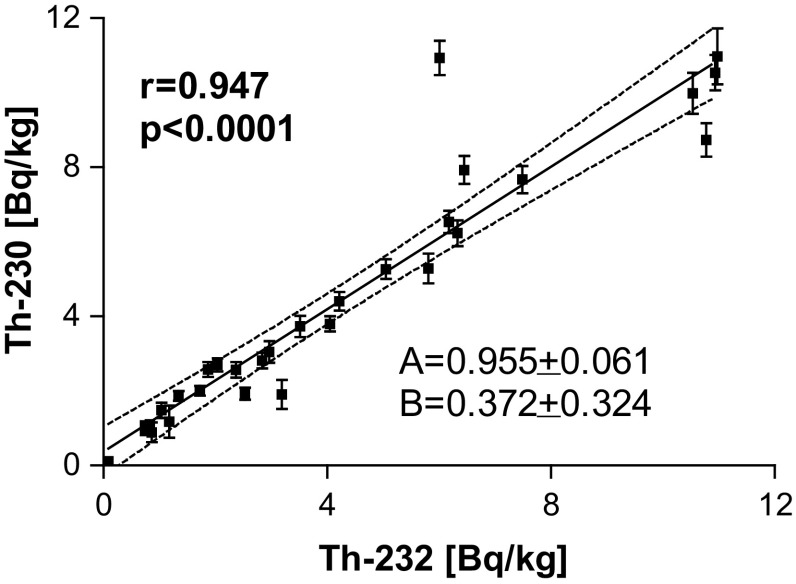
Correlation between thorium isotopes activities. Despite a big difference in activities between the samples, the isotopes from uranium series (^230^Th) and from thorium series (^232^Th) are well correlated, *r* is Pearson’s correlation factor, *p* is the significance level, A and B are slope and intercept of correlation line, respectively

Results for thorium activities are presented in Table 6. They were calculated under assumption of complete, equal to 100%, recoveries of thorium. They vary a lot, ranging (for ^232^Th) from 0.09 ± 0.01 Bq/kg (Białe Wigierskie Lake) to almost 11 Bq/kg for a couple of lakes like Aszarynis, Szelment, Sumowo, Sunnowo. Despite a big difference in activities between the samples, isotopes from uranium series (^230^Th) and from thorium series (^232^Th) are very well correlated (see Fig. 2). The Persons correlation factor *r* = 0.947 with significance level *p* < 0.0001. Thorium isotopes are in almost constant proportion, close to one to one for all samples but few, namely those of Dobskie and Pobojno Lakes, which are significantly above the correlation line and Aszarynis and Dębniak Lakes, which are significantly below the correlation line. This results suggests almost uniform geological structure of examined bottom sediments. Two samples from one lake (Sumowo) lake had identical thorium isotopes activity and almost identical ^239+240^Pu activity, but they differ each to other for ^238^Pu activity. Since the main source of this isotope is Chernobyl, it might be caused by scattered, particle nature of Chernobyl Pu fallout.

**Table 6:** Results on Th activity in lakes sediments calculated under assumed complete, equal to 100% recoveries of thorium, real value are at least those presented

Name	^232^Th (Bq/kg)	^230Th^ (Bq/kg)
	A ± dA	A ± dA
Aszarynis	10.78 ± 0.49	8.74 ± 0.45
Białe Filipowskie	1.18 ± 0.43	1.18 ± 0.43
Białe Wigierskie	0.09 ± 0.01	0.12 ± 0.01
Brożane	2.53 ± 0.16	1.93 ± 0.16
Dębniak	1.87 ± 0.17	2.58 ± 0.20
Dobskie	6.01 ± 0.46	10.94 ± 0.46
Gaładuś	4.05 ± 0.21	3.80 ± 0.20
Garbaś	0.74 ± 0.09	0.92 ± 0.10
Guzki	3.52 ± 0.28	3.73 ± 0.29
Jałówek	5.81 ± 0.41	5.28 ± 0.40
Jałowo	4.22 ± 0.24	4.40 ± 0.25
Jędzelewo	1.34 ± 0.11	1.86 ± 0.13
Krzywe	2.84 ± 0.20	2.82 ± 0.21
Lepaki Wielkie	2.96 ± 0.29	3.05 ± 0.29
Oleckie Wielkie	0.86 ± 0.27	0.89 ± 0.27
Płaskie	2.04 ± 0.16	2.70 ± 0.17
Pobojno	6.45 ± 0.35	7.93 ± 0.37
Rajgrodzkie	1.72 ± 0.12	2.01 ± 0.13
Rogale Duże	3.19 ± 0.39	1.91 ± 0.39
Sawinda Wielka	0.74 ± 0.09	1.07 ± 0.09
Serwy	0.83 ± 0.09	1.10 ± 0.10
Sumowo 2	10.94 ± 0.48	10.54 ± 0.48
Sumowo 1	10.98 ± 0.76	10.97 ± 0.75
Sunnowo	7.49 ± 0.37	7.67 ± 0.37
Szelment Wielki	10.54 ± 0.55	9.99 ± 0.55
Tajty	2.37 ± 0.18	2.57 ± 0.21
Tobołowo	5.05 ± 0.27	5.27 ± 0.27
Toczyłowo	1.04 ± 0.16	1.48 ± 0.20
Wiżajny	6.33 ± 0.35	6.23 ± 0.34
Zawadzkie	6.18 ± 0.30	6.53 ± 0.30
IAEA 300	25.39 ± 1.23	35.02 ± 1.28

**Fig. 3:**
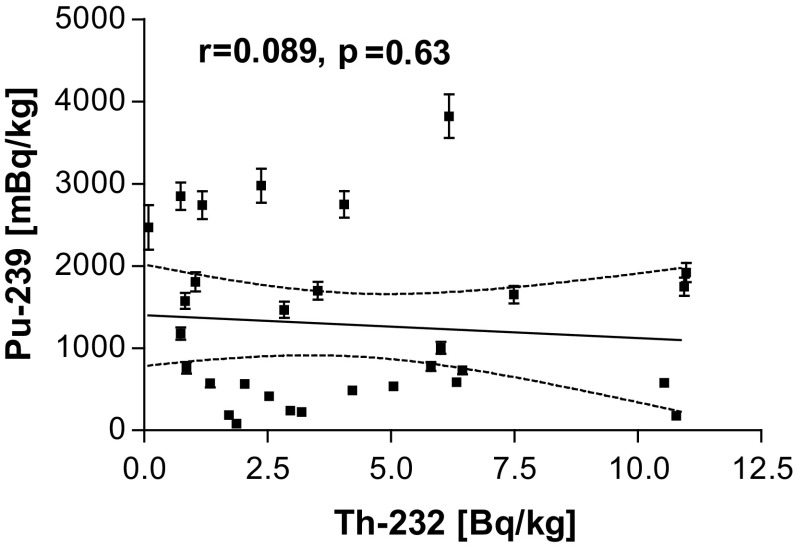
Correlation plot for the activities of ^232^Th and ^239+240^Pu in lake sediment samples, a lack of correlation observed

There is a complete lack of correlation between ^232^Th and ^239+240^Pu (see Fig. 3), what might suggests, that the origin of radionuclides (which is different for those nuclides) but not the accumulation properties of sediments (expected similar for both radionuclides) is the main factor which governs the observed variety of activity levels.

**Fig. 4:**
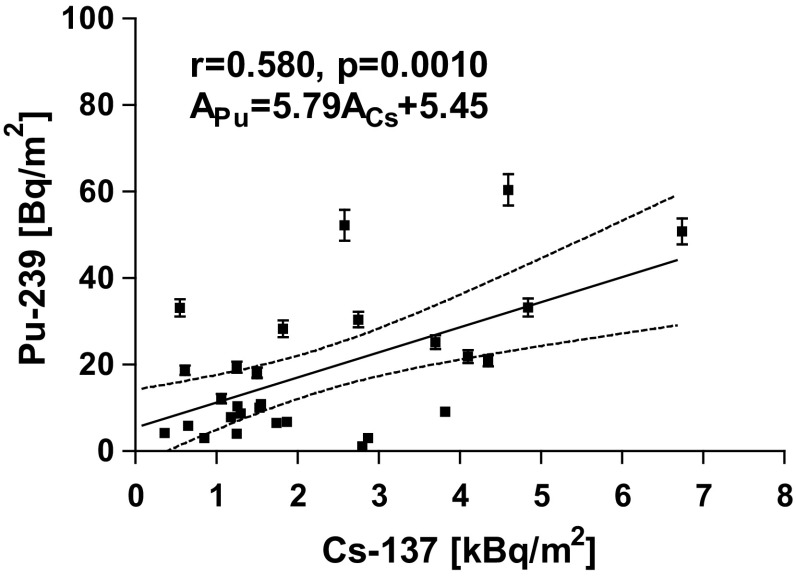
Correlation plot for ^137^Cs and ^239+240^Pu surface activity in bottom sediment of lake samples

**Fig. 5:**
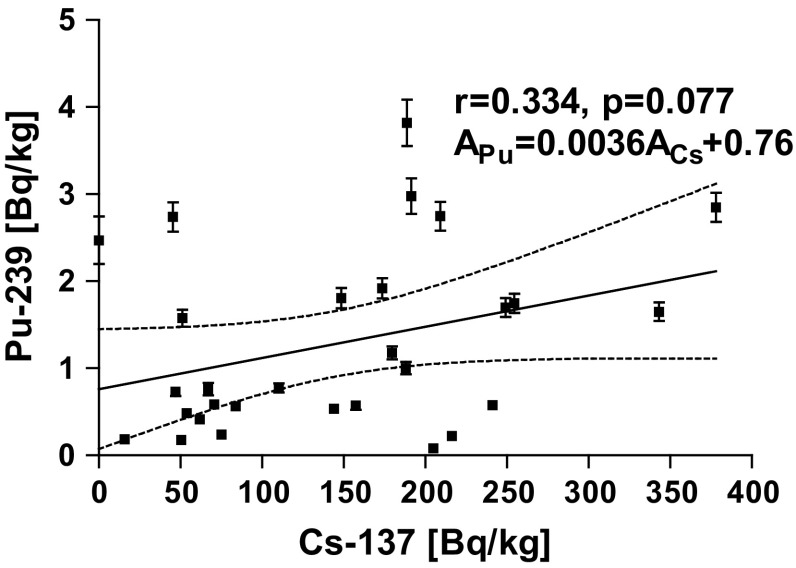
Correlation plot for ^137^Cs and ^239+240^Pu activity concentration in bottom sediment of lake samples

A significant correlation was found for surface activity between the activity of radiocaesium determined previously [5] and ^239+240^Pu (Fig. 4). The Persons correlation factor r was equal to 0.582 with the level of significance *p* = 0.001. Less significant (*r* = 0.337, *p* = 0.077) correlation calculated for activity concentrations of those radionuclides (Fig. 5). Low correlation characterized by *R* = 0.350 and *p* = 0.068 was found between the pH in sediment measured in water and the ^239+240^Pu in sediment (Fig. 5). Perhaps it is caused by a higher mobility of humic acid in more basic environment. The humic acid is well known complexing agent for plutonium [15, 16]. No correlation was found between the Pu content and basic matrix content of samples like: SiO_2_, Ca, Mg, Na, K, C, P, Fe.

In principle, like for radiocaesium, differentiation of plutonium deposition in the lake bottom sedimentations may be due to the morphometric parameters of the lakes and the type of the drainage area. A differentiation has been made on the basis of the trophic levels of lakes. It was found previously for radiocesium [5] in present samples that activity of the poorest oligotrophic lakes have shown the lowest value of deposition with AM equal to 1.49 kBq/m^2^, whereas richer mesotrophic and eutrophic lakes have been found to have feature higher depositions of AM = 2.23 kBq/m^2^ and 1.86 kBq/m^2^, respectively. For ^239+240^Pu no such a feature was observed. Arithmetic mean values were 22.0 Bq/m^2^, 25.9 Bq/m^2^ and 14.1 Bq/m^2^ for oligotrophic, mesotrophic and eutrophic lakes, respectively. The difference in deposition observed in examined layer of bottom sediment between caesium and plutonium might be caused by a difference of the scale of biological reflux of radionuclides, which apparently seems to be higher for caesium than for plutonium.

## Conclusions

In average about 30% or 40% of expected amount of global fallout plutonium was observed in examined 2 cm slice of top layer of bottom sediment from 29 Mazurian lakes. Chernobyl plutonium was not so much abundant as was expected from previous investigations conducted on this area using forest litter samples, reaching maximum content of 24%. Moreover, later performed analyses of 10 sediments from lakes, where deeper layers were also available did not confirmed initial analyses revealing even lower Chernobyl content and indicating problems with homogenity of Pu content in bottom sediments. This was result mainly of very wet, sludge form.

This difference between forest litter and lake sediment seems to be the most interesting observation of present work. One of possible explanation is the difference in the aerodynamic properties of forest and lake which might influence the intensity of hot particle fallout.

Unlikely the radiocaesium [5], the plutonium content in top layer sediment of lakes seems to have in presented here cases no clear relationship with the trophic status of lake. However, a weak but significant correlation between plutonium and cesium surface activity was noticed. Hardly no correlation was observed between plutonium activity and any of known for given lake parameter, among them such like main content of matrix. Only a weak correlation was found between Pu content and pH of sediment determined in water.
